# Acute Severe Transaminitis as a Unique Presentation of Chronic Cholecystitis

**DOI:** 10.7759/cureus.16102

**Published:** 2021-07-02

**Authors:** Huda Fatima, Deepti Avasthi

**Affiliations:** 1 Internal Medicine, St. Vincent Mercy Medical Center, Toledo, USA

**Keywords:** chronic cholecystitis, cholestatic liver injury, gallstone cholecystitis, transaminitis, liver function tests (lfts), elevated liver-associated enzymes

## Abstract

The hepatocellular function can be evaluated using aspartate aminotransferase (AST) and alanine aminotransferase (ALT) which are biochemical markers of the liver. Whenever there is an ischemic, toxic, or inflammatory injury to the liver, necrosis of the hepatocytes occurs and these biochemical markers are released into the circulation, showing an acute elevation in serum levels.

In this case report, we discuss the unique clinical presentation of a female patient who came to the Emergency Room (ER) with acute onset chest pain with laboratory findings of elevated serum aminotransferases and cholestatic markers and was ultimately diagnosed with chronic cholecystitis.

The usual clinical presentation associated with extremely elevated levels of liver enzymes can be one of three cases: acute viral hepatitis, toxin-induced liver injury, or acute ischemic insult to the liver. However, our patient was diagnosed with chronic cholecystitis despite her unique initial presentation of acute, severe transaminitis.

While one may find elevated liver enzyme levels in acute cholecystitis, owing to the sudden nature of the inflammatory process, chronic cholecystitis is not known to cause high levels of serum amino transaminases or fulminant liver failure.

Our case report indicates a diverse phenotype of chronic cholecystitis with an unusual presentation of acute, severe transaminitis. It helps expand the differential diagnoses of acute elevation of liver function tests (LFTs). Further studies are needed to explore the pathology behind chronic cholecystitis in order to understand its impact on liver damage.

## Introduction

The most common markers of hepatocellular function are aspartate aminotransferase (AST) and alanine aminotransferase (ALT). Hepatocyte necrosis in acute hepatitis, ischemic, or toxic injury of the liver results in their leakage into the blood circulation and acute elevation of serum levels of these liver enzymes. Cholestasis (defined as a lack of bile flow) results from the blockage of bile ducts or from diseases that impair bile formation in the liver itself. Alkaline phosphatase (ALP) and gamma glutamyl-transferase (GGT) levels usually rise to several times the normal range after some days of bile duct obstruction or intrahepatic cholestasis, thereby supporting the diagnosis of gallstone disease [1]. However, there are very few studies on the impact of gallstone-related disorders on liver function tests and their results are inconsistent so far [2].

Herein, we share an interesting case report of a patient who presented to the emergency room (ER) within a few hours of chest pain and was found to have acutely elevated liver enzymes, alongside moderate elevations of markers of cholestasis, and was diagnosed with chronic cholecystitis.

## Case presentation

The patient was a 65-year-old Caucasian female, who presented with acute onset substernal chest pain which woke her up from sleep. The pain was radiating to the left shoulder, pressure-like in nature, and associated with shortness of breath and diaphoresis. The patient gave a history of having shoveled snow one day prior to the onset of pain, but there were no other aggravating or relieving factors. She had a past medical history significant for hypertension, anxiety, gastroesophageal reflux disease (GERD), and a past surgical history significant for left knee arthroplasty. She denied any history of smoking, alcohol use, or drug use, including herbal and homeopathic or over-the-counter medications. The patient did not have any significant family history. Cardiac workup was negative, and the patient’s labs were significant for elevated ALT, AST, ALP, bilirubin with elevated direct bilirubin, and elevated GGT levels (Table 1). Urine and drug toxicology screens were negative, including for serum acetaminophen and salicylate levels, as shown in Table 1.

**Table 1:** Liver Function Test Results ALP: alkaline phosphatase; ALT: alanine aminotransferase; AST: aspartate aminotransferase; GGT: gamma glutamyl-transferase

	ALT	AST	ALP	Bilirubin	Direct bilirubin	GGT	Serum acetaminophen	Serum salicylate
	1,311	1,206	201	2.20	1.27	321	< 5	1
Reference range	5 - 33 U/L	< 32 U/L	35 - 104 U/L	0.3 -1.2 mg/dL	< 0.31 mg/dL	5 - 36 U/L	10 - 30 ug/ml	3 - 10 mg/dL

On physical examination, the patient was alert and oriented to time, place, and self, with no symptoms of even mild encephalopathy. There was slight epigastric tenderness on palpation, but no findings of scleral icterus, jaundice, positive Murphy’s sign, or stigmata of liver disease were present. Due to concern for acute liver injury due to such markedly elevated liver enzymes, an acute hepatitis panel was ordered to rule out viral etiology, including hepatitis A, B, and C, as shown in Table 2.

**Table 2:** Viral Hepatitis Test Results IgM: immunoglobulin M

Viral Hepatitis Panel	Results
Hepatitis A IgM	Nonreactive
Hepatitis B surface antigen	Nonreactive
Hepatitis C Antibody	Nonreactive
Hepatitis B core antibody IgM	Nonreactive

A lipid panel was ordered which was within normal limits, thus ruling out non-alcoholic fatty liver disease (Table 3).

**Table 3:** Lipid Panel Test Results HDL: high-density lipoprotein; LDL: low-density lipoprotein

Lipid panel	Results	Reference ranges
Total cholesterol	152	< 200 mg/dL
LDL cholesterol	77	0 - 130 mg/dL
HDL cholesterol	67	> 40 mg/dL
Triglycerides	42	< 150 mg/dL
Cholesterol/HDL ratio	2.3	< 5

Careful evaluation of the patient’s home medications and medication history was done to check for drug-associated liver injury (DILI). Her daily home medications included citalopram 20 mg, multivitamins, vitamin B12, potassium chloride, lorazepam 1 mg, omeprazole 20 mg daily, and vitamin D3 125 mcg. While omeprazole and citalopram can both be a potential source of DILI, there was no record of previous clinical or laboratory abnormalities, no recent changes to the dosing nor were either of these medications recently started by the patient’s primary care physician, which opposed the diagnosis of DILI being the source of these acute elevations in serum transaminases.

Autoimmune workup was initiated and serum anti-neutrophilic antibody, anti-smooth muscle antibody, liver-kidney microsomal antibody, serum alpha-1-anti trypsin antibody, and serum ceruloplasmin were all tested. Thus far, all workups remained within normal limits, thus ruling out differentials such as primary biliary cirrhosis, alpha 1 anti-trypsin deficiency, and Wilson disease (Table 4). The only positive lab finding was a significantly elevated serum ferritin level of 1,710 ug/L (reference range: 13 - 150 ug/L), which could be a potential indicator of hemochromatosis. However, the patient had no other clinical manifestations, such as diabetes, jaundice, or arthralgias.

**Table 4:** Autoimmune Test Results

Antibodies	Results	Reference range
Anti-neutrophilic antibody	Negative	
Liver-kidney microsomal antibody	< 1.20	< 1.20
Anti-mitochondrial antibody	1.3	0.0 - 4.0 U/ml
Anti-smooth muscle antibody	4	0 - 19 units
Alpha 1-antitrypsin antibody	166	90 - 200 mg/dL

Table 5 below shows the patient’s LFT progression during her hospital stay.

**Table 5:** Progression of Patient's Liver Function Tests During Hospital Stay ALP: alkaline phosphatase; ALT: alanine aminotransferase; AST: aspartate aminotransferase

Date	Albumin	ALP	ALT	AST	Bilirubin
Reference Ranges:	3.5 - 5.2 g/dL	35 - 104 U/L	5 - 33 U/L	< 32 U/L	0.3 - 1.2 mg/dL
2/17/21	4.0	201	1,311	1,206	2.20
2/18/21	3.7	214	1,507	876	4.86
2/19/21	3.7	196	968	279	1.55
2/20/21	3.4	169	521	192	1.05
2/21/21	3.5	175	395	65	0.94
2/22/21	3.5	129	265	38	0.75
2/23/21	3.6	117	227	55	0.65
2/24/21	3.7	97	248	106	0.62

Throughout the patient’s inpatient admission, her international normalized ratio (INR) remained between 0.9 - 1.0, with no coagulopathy or overt bleeding noted. No portal hypertension was noted on imaging and the patient remained on prophylactic anticoagulation for deep venous thrombosis with no break in between.

**Figure 1:**
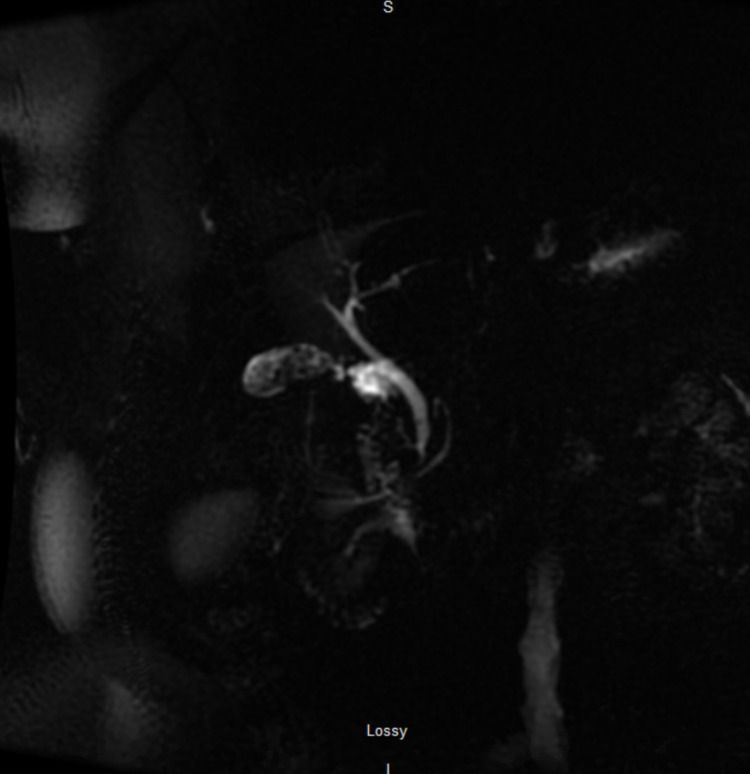
MRCP of the abdomen, radial view Cholelithiasis was noted without features of acute cholecystitis, i.e., gallbladder wall thickening or pericholecystic fluid. The 2 - 3 mm filling defect at the distal common duct is in keeping with a gallstone. MRCP: magnetic resonance cholangiopancreatography

Initial imaging included a liver ultrasound which showed increased echogenicity without evidence of intrahepatic biliary ductal dilatation and normal hepatopetal flow. The gallbladder contained mobile stones but no gallbladder wall thickening or pericholecystic fluid was seen. Murphy's sonographic sign was negative. The common bile duct (CBD) diameter was within the upper limits of the normal range, measuring 6.8 mm. A magnetic resonance cholangiopancreatography (MRCP) with and without contrast was completed. This study showed cholelithiasis without MR features of acute cholecystitis and a single small stone at the distal common duct with no intra or extrahepatic bile duct dilatation (Figure 1). No common bile duct stricture was evident. Hepatic steatosis was noted; no other discrete lesion was evident.

**Figure 2:**
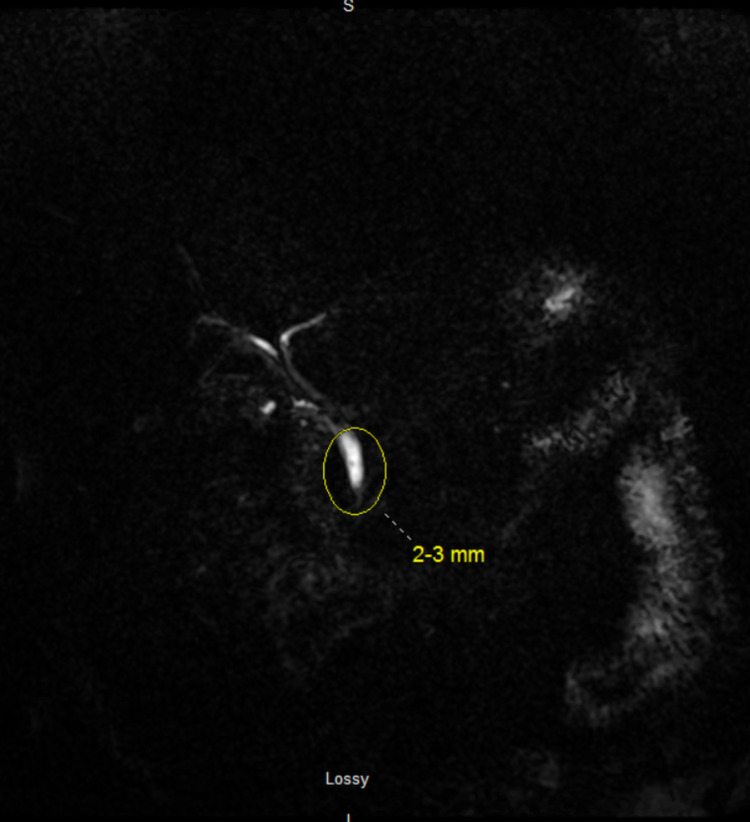
MRCP of the abdomen, coronal view The 2 - 3 mm filling defect at the distal common duct demonstrates the presence of a single small stone with no intra or extrahepatic bile duct dilatation. No common bile duct stricture was evident. MRCP: magnetic resonance cholangiopancreatography

Figure 2 shows a single stone at the distal common duct without any intrahepatic or extrahepatic ductal dilatation. 

A plan was made to proceed with an endoscopic retrograde cholangiopancreatography (ERCP). During the ERCP, a biliary sphincterotomy was performed, and the CBD stone was removed. Sludge and debris extraction was completed from the CBD and the intrahepatic biliary duct. A cholangiogram was performed during which the gallbladder and the cystic duct did not fill with contrast; therefore, a plastic biliary stent was placed.

**Figure 3:**
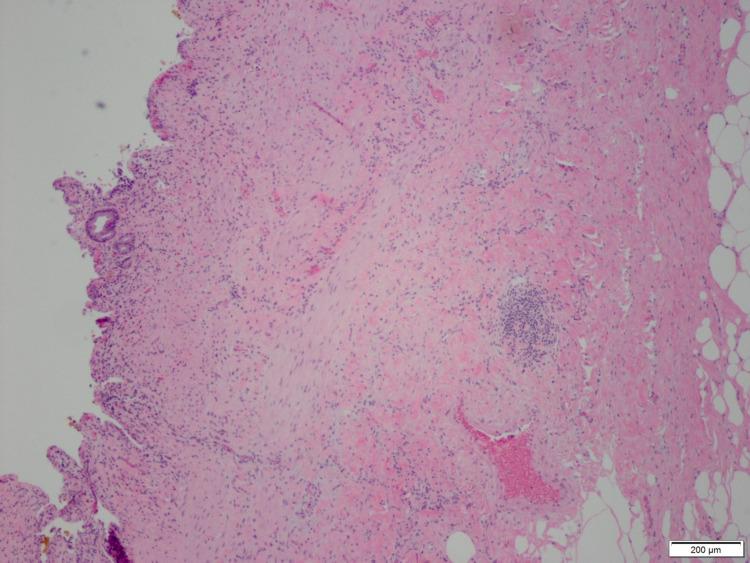
Histological slide of gallbladder specimen The gallbladder wall is thickened with infiltration by predominantly mononuclear leukocytes with a sprinkling of neutrophils. Near the surface, small aggregates of bile are present but no bile granulomas are identified.  Fibromuscular hypertrophy and fibrosis characterize the wall.  Much of the mucosa is denuded but no lamina propria foamy histiocytes are present. No mural xanthomas or granulomas are present.

The recommendation was made for a surgical consult for cholecystectomy for what was considered to be a clinical presentation of "early acute cholecystitis." The patient underwent laparoscopic, robotic-assisted, fenestrated subtotal cholecystectomy which showed a contracted gallbladder, omental adhesions, and gall bladder wall thickening but no wall edema, signs which indicated a chronic inflammation of the gall bladder. The surgical pathology report described the gross specimens as gallbladder fragments 4.0 x 2.5 x 2.0 cm in aggregate, with identifiable serosal surfaces being pink-tan and the liver bed being coarse tan-brown. The gall bladder wall was 0.1 cm in thickness and the mucosa was tan pink and focally granular. The gallstones were described as fragmented yellow calculi 2.5 x 2.5 x 1.0 cm in aggregate. An image of the histological slide is shown in Figure 3 below.

The postoperative diagnosis was choledocholithiasis and chronic cholecystitis. The patient tolerated the procedure safely and was discharged to home within 48 hours with follow-up planned with gastroenterology for biliary stent removal and with general surgery for postoperative care.

## Discussion

The interesting manifestation of this clinical presentation was the extremely elevated levels of the liver enzymes, ALT and AST, which were found to be in the 1,000s. Such high aminotransferase levels are usually found in one of three cases: an acute ischemic insult to the liver, toxin-induced liver injury, or acute viral hepatitis. However, our patient ultimately had a histological and surgical diagnosis of chronic cholecystitis despite her unique initial presentation of acute severe transaminitis.

Chronic cholecystitis is a histological diagnosis. It is characterized by the presence of chronic inflammatory cell infiltration of the gallbladder. The pathophysiology of chronic cholecystitis is poorly understood, although it is considered to be the indication for almost 3.0% of cholecystectomies in adult patients worldwide [3]. The most common explanation is that of an evolving inflammatory process with recurring episodes of low-grade gallbladder obstruction, resulting in repeated mucosal trauma and acute inflammation. As each episode of acute inflammation resolves, the neutrophils are replaced by lymphocytic, plasma cells, and macrophage infiltration. Focal ulcerations and necrotic deposits are replaced by collagen and granulation tissue, and the gall bladder wall is generally found to be thickened and fibrotic. Its chronic presentation differentiates it from acute cholecystitis, although the clinical presentation is varied, ranging from asymptomatic incidental findings to abdominal pain, biliary colic, obstructive jaundice, or even a palpable mass. Another unique variant of chronic cholecystitis is xanthogranulomatous cholecystitis, which is histologically characterized by a focal or diffuse destructive inflammatory process, followed by marked proliferative fibrosis and infiltration of lipid-laden macrophages and foamy cells [4]. It is proposed to be the result of a rupture of the Rokitansky-Aschoff sinuses or mucosal ulceration due to increased intraluminal pressure secondary to duct obstruction, which then leads to the entry of bile in the gallbladder wall. This intramural bile is then partially engulfed by the macrophages, leading to a chronic granulomatous inflammatory response [4]. However, in this case report, the histological diagnosis did not show any lipid-laden macrophages, thereby ruling out xanthogranulomatous cholecystitis. The degree of gallbladder wall inflammation is poorly correlated to the number or volume of gall stones, with up to 12% - 13% of patients with chronic cholecystitis demonstrating no choleliths on presentation [5].

While liver enzyme tests are routinely performed on all patients presenting with symptomatic gallstone disease, a few studies have assessed the impact of gallstone-related disorders on LFTs or the role of these tests in acute inflammatory gallstone disease and have reported varied and inconclusive results [2]. Chronic cholecystitis is not known to cause high elevations of liver enzymes or cause fulminant liver failure. In fact, it is acute cholecystitis in which one may find deranged liver enzyme levels, owing to the sudden nature of the inflammatory process but rarely in chronic gallbladder inflammation. Diagnostic confusion can occur when a patient presents within a few hours of acute bile duct obstruction secondary to a gallstone. In such a situation, there may be an acute rise in ALT and AST levels up to 500 U/L or more in the first few hours followed by a decline, whereas there is a longer duration of time for ALP and GGT to rise [1]. One study in 2005 showed GGT to be a highly specific and sensitive marker for the diagnosis of a CBD stone in acute, chronic, or acute-on-chronic cholecystitis, but there has been scarce substantiation in the literature to support serum aminotransferase elevations as a valid marker suggestive for gallstone disease [5]. The presence of chronic cholecystitis as a possible risk factor for liver disease appears to be an understudied topic overall. A recent study showed a high number of inflamed and/or necrotic-appearing gallbladders in liver transplant recipients at the time of transplantation, with 28% of the patient population in this study shown to have underlying chronic cholecystitis [6]. This study revealed cholecystitis to be under-recognized as an independent risk factor affecting the outcomes of the high-model end-stage liver disease (MELD) population undergoing liver transplantation.

## Conclusions

Our case report presents an unusual but important feature of chronic cholecystitis and indicates a diverse phenotype of this disease. The case report is helpful in expanding the differential diagnoses of acute elevation of LFTs as it adds to the evidence that it is possible for a clinical presentation of chronic cholecystitis to include high levels of aminotransferases. It provides an affirmative answer to the question: are LFTs definitively useful in identifying patients with inflammatory gallstone disease? It also raises a debate about the value of elevation in liver enzymes in gallbladder diseases and the need for careful interpretation of these tests when evaluating etiology for acute liver failure. Further studies are indicated to explore the pathology behind chronic cholecystitis to understand its impact on liver damage.
